# P-21. Comparison of Three RSV Vaccine Lower Respiratory Tract Disease Primary Endpoint Definitions for Adult Vaccine

**DOI:** 10.1093/ofid/ofae631.229

**Published:** 2025-01-29

**Authors:** Sarah E Williams, Elizabeth Begier, Kumar Ilangovan, Cassandra Hall-Murray, Bradford D Gessner

**Affiliations:** Pfizer Vaccines, Nashville, Tennessee; Pfizer Vaccines, Nashville, Tennessee; Vaccine Research and Development, Pfizer, USA, Raleigh, North Carolina; Pfizer, Inc., Collegeville, Pennsylvania; Pfizer Biopharma Group, Collegeville, Pennsylvania

## Abstract

**Background:**

Despite their public health importance, a consensus definition for lower respiratory tract disease (LRTD), or illness (LRTI) is lacking. Clinical trials of three respiratory syncytial virus (RSV) vaccines for older adults used different LRTI/LRTD definitions for vaccine efficacy (VE). We hypothesize differences between definitions could bias product comparisons.

**Table 1.**
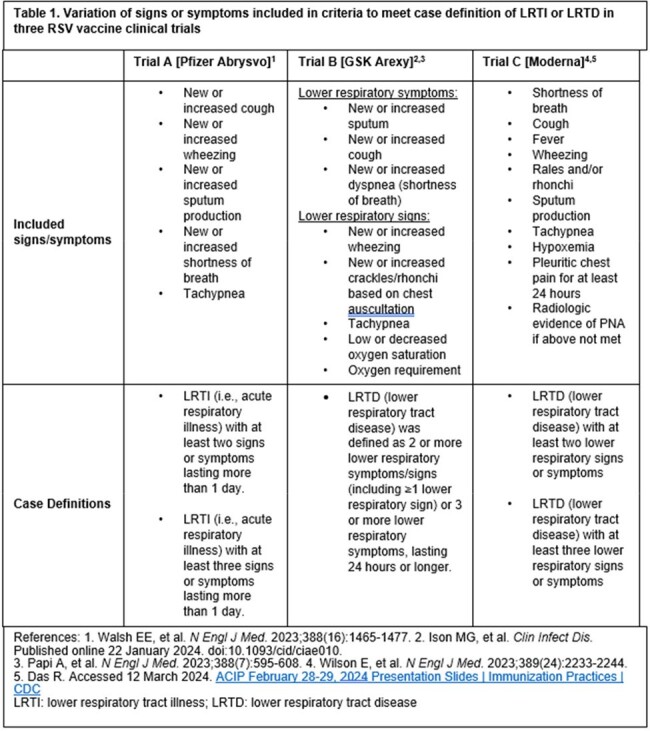
Variation of signs or symptoms included in criteria to meet case definition of LRTI or LRTD in three RSV vaccine clinical trials

**Methods:**

We reviewed case definitions for LRTI/LRTD in RSV vaccine clinical trials conducted in older adults.

**Table 2.**
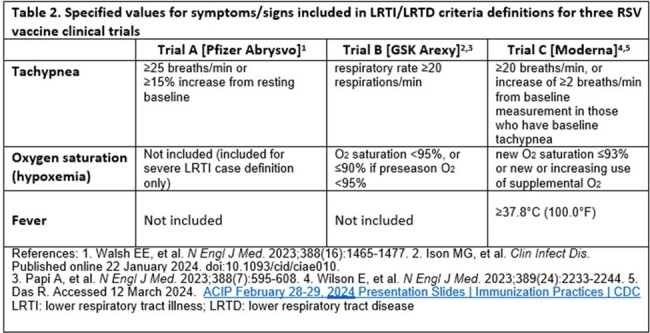
Specified values for symptoms/signs included in LRTI/LRTD criteria definitions for three RSV vaccine clinical trials

**Results:**

Two of three published RSV vaccine clinical trials from Pfizer and Moderna used two outcomes (LRTI/LRTD with ≥2 signs/symptoms or ≥3 signs/symptoms) to measure VE, while the third trial from GlaxoSmithKline (GSK) used one (combining ≥2 and ≥3 signs/symptoms) (Table 1). The Pfizer trial included 5 signs/symptoms as components of LRTI; three of these (tachypnea, wheezing, shortness of breath) suggest lower airway involvement and two (cough and self-reported sputum production) could also result from upper airway infection. At interim analysis, 29 of 45 cases had only 2 symptoms (64.4%). Of these 29, 25 had only cough and sputum production (86.2%). The GSK trial, by contrast, required all LRTD cases to include at least one sign, all of which indicated lower airway involvement (Table 2). The Moderna trial included 10 signs/symptoms, including the potential for subjects to have only cough and sputum production. Even within signs/symptoms, definitions differed (e.g., tachypnea cutoff ranged from 20 to 25 breaths per minute; Table 2). When using comparable definitions, full season 1 VEs for Pfizer and GSK products were similar while the point estimate for the Pfizer product was higher during season 2 (Table 3).

**Table 3.**
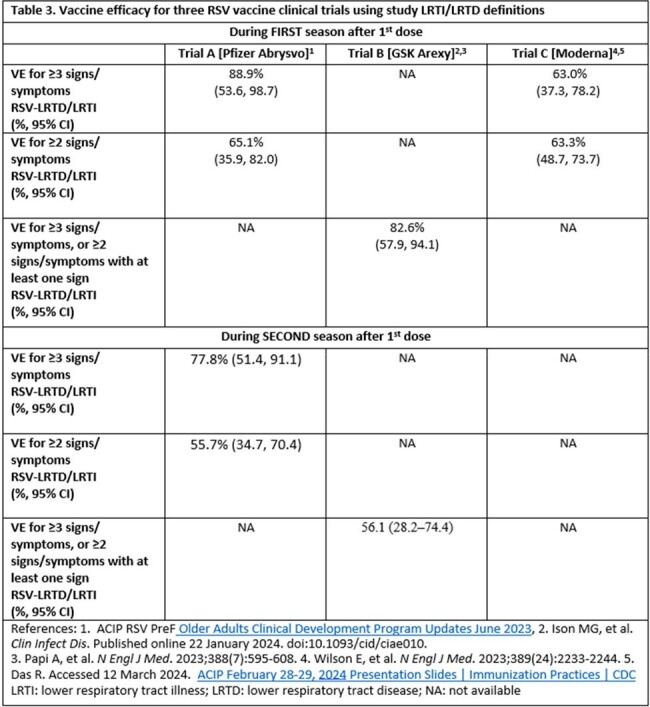
Vaccine efficacy for three RSV vaccine clinical trials using study LRTI/LRTD definitions

**Conclusion:**

Lack of standard case definitions limit ability to compare results across trials. Some ≥2 LRTI events for Pfizer and Moderna may not have involved the lower respiratory tract but rather the upper respiratory tract only. The most comparable case definitions likely are ≥3 signs/symptoms LRTI in the Pfizer and Moderna trials, and LRTD in the GSK trial.

**Disclosures:**

**Sarah E. Williams, MD, MPH**, Pfizer, Inc: Salary|Pfizer, Inc: Stocks/Bonds (Private Company) **Elizabeth Begier, MD, M.P.H.**, Pfizer Vaccines: Employee|Pfizer Vaccines: Stocks/Bonds (Private Company) **Kumar Ilangovan, MD, MSPH, MMCi**, Pfizer, Inc.: salary|Pfizer, Inc.: Stocks/Bonds (Public Company) **Cassandra Hall-Murray, PharmD**, Pfizer, Inc.: employee|Pfizer, Inc.: Stocks/Bonds (Public Company) **Bradford D. Gessner, M.D., M.P.H.**, Pfizer: Employee|Pfizer: Stocks/Bonds (Public Company)

